# Identifying the culprit artery via 12‐lead electrocardiogram in inferior wall ST‐segment elevation myocardial infarction: A meta‐analysis

**DOI:** 10.1111/anec.13016

**Published:** 2022-11-01

**Authors:** Peng Zhou, Yingying Wu, Meng Wang, Yikai Zhao, Yangjie Yu, Maieryemu Waresi, Huiyang Li, Bo Jin, Xinping Luo, Jian Li

**Affiliations:** ^1^ Department of Cardiology Huashan Hospital, Shanghai Medical College, Fudan University Shanghai China; ^2^ Department of Endocrinology and Metabolism Huashan Hospital, Shanghai Medical College, Fudan University Shanghai China

**Keywords:** culprit artery, electrocardiogram, inferior wall, ST‐segment elevation myocardial infarction

## Abstract

**Background:**

Inferior wall ST‐segment elevation myocardial infarction (STEMI) is mostly caused by acute occlusion of right coronary artery (RCA) and left circumflex artery (LCX). Several methods and algorithms using 12‐lead ECG were developed to localize the lesion in inferior wall STEMI. However, the diagnostic properties of these methods remain under‐recognized.

**Aims:**

The aim of this meta‐analysis is to compare the diagnostic properties among the methods of identifying culprit artery in inferior wall STEMI using 12‐lead ECG.

**Methods:**

We performed a meta‐analysis to calculate the pooled sensitive, specificity, area under the curve (AUC) and diagnostic odds ratio (DOR) of each method.

**Results:**

Thirty‐three studies with 4414 participants were included in the analysis. Methods using double leads had better diagnostic properties, especially ST‐segment elevation (STE) in III > II [with pooled sensitivity 0.89 (0.84–0.93), specificity 0.68 (0.57–0.79), DOR 17 (9–32), AUC 0.88 (0.85–0.91)], ST‐segment depression (STD) in aVL > I [with pooled sensitivity 0.82 (0.72–0.90), specificity 0.69 (0.48–0.86), DOR 11 (4–29), AUC 0.85 (0.81–0.88)], and STD V3/STE III ≤1.2 [with pooled sensitivity 0.88 (0.78–0.95), specificity 0.59 (0.42–0.75), DOR 12 (5–27), AUC 0.82 (0.78–0.85)]. Diagnostic algorithms, including Jim score[pooled sensitivity 0.70 (0.55–0.85), specificity 0.88 (0.75–0.96)], Fiol's algorithm [pooled sensitivity 0.54 (0.44–0.62), specificity 0.92 (0.88–0.96)] and Tierala's algorithm [pooled sensitivity 0.60 (0.49–0.71), specificity 0.91 (0.86–0.96)], were not superior to these simple methods.

**Conclusions:**

Our meta‐analysis indicated that diagnostic methods using double leads had better properties. STE in III > II together with STD in aVL > I may be the most ideal method, for its accuracy and convenience.

## Graphical abstract

Sometimes the easiest way is the best. Simple methods as ST‐segment elevation in III > II and ST‐segment depression in aVL > I are effective diagnostic test for identifying culprit artery in inferior wall ST‐segment elevation myocardial infarction. Complex algorithms are not superior to simple methods.

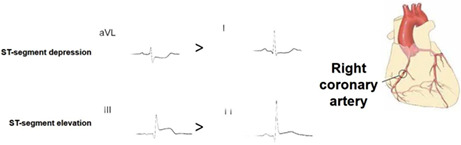

## 1 INTRODUCTION

Inferior wall ST‐segment elevation myocardial infarction (STEMI) is estimated to be 40%–50% of all STEMIs, with a mortality of 2%–9% (Aguiar et al., 2018). It is mostly caused by acute occlusion of right coronary artery (RCA) and left circumflex artery (LCX) (Patel, 2008). With a different culprit artery, patients can exhibit different complications and prognosis (Karwowski et al., 2017), as complete atrioventricular block (AVB) is more common in patients with proximal RCA lesion with higher mortality (Jim et al., 2010). Early Identification of culprit artery may help in the treatment of inferior wall STEMI.

It is recommended to initiate electrocardiogram (ECG) monitoring as soon as possible in all patients with suspected STEMI in guidelines (Ibanez et al., 2018; Levine et al., 2016). ST‐segment elevation in lead II, III and aVF predicts of the presence of RCA or LCX disease, regardless of whether a right, left or balanced distribution is present (Bhatt et al., 2022; Fuchs et al., 1982). However, it is hard to distinguish RCA disease from LCX disease by ECG criteria (Fuchs et al., 1982). There were a number of studies in last three decades on different ECG findings between inferior wall STEMI caused by RCA and LCX (Almansori et al., 2010; Bairey et al., 1987; Bayram & Atalay, 2004; Chia et al., 2000; Gaballa et al., 2019; Huang et al., 2016; Li et al., 2011; Li et al., 2017; Ruiz‐Mateos et al., 2020; Sun et al., 2007) and these researchers proposed several methods and algorithms (Fiol et al., 2004; Jim et al., 2012; Kosuge et al., 1998). Nevertheless, the diagnostic properties of these methods remain under‐recognized. Therefore, we conducted a meta‐analysis for previous studies on culprit artery identification in inferior wall STEMI using ECG, to assess the diagnostic properties of these methods and algorithms.

## 2 METHODS

### 2.1 Search strategy

We followed the Preferred Reporting Items for Systematic reviews and Meta‐Analyses (PRISA) 2020 checklist. The protocol submitted to PROSPERO was still under review (PROSPERO ID 330858). Electronic databases were used for relevant studies, and EMBASE, PubMed, Web of Science, and Cochrane databases were referred systematically for studies that were published up to April 2022. Mesh terms were applied as search terms, including “ST Elevation Myocardial Infarction” and “electrocardiogram.”

### 2.2 Study selection and quality assessment

We enrolled the studies as the following criteria. (1) Both retrospective and prospective studies on culprit artery identification in inferior wall STEMI using ECG (the index test), with coronary angiography (CAG) as reference standard. (2) Studies providing complete data or adequate data for calculating the number of true positive, false positive, false negative and true negative of each diagnostic method. The exclusion criteria were as follows. (1) Abstract without detailed results, reviews or systemic reviews. (2) Patients with prior myocardial infarction, history of coronary artery bypass grafting (CABG). (3) Studies focusing on patients with chronic coronary total occlusion (CTO) lesion in non‐culprit arteries. In case of various publications, the recent studies or studies of larger population were used.

In these studies, the 12‐lead ECG was recorded at standard paper speed of 25 mm/s and calibration of 10 mm/mV. Diagnostic criteria of inferior wall STEMI: myocardial ischemic symptoms; new onset of two or three leads of ST‐segment elevation (STE) > 1 mm in II, III, AVF recorded within 12 h form the onset of symptom; and an appropriate rise and fall of creatine kinase and troponin I/T levels. In other leads, ST‐segment depression (STD) was considered significant when ≥0.5 mm. Any STE was considered significant in other leads.

Citations were selected after initially screening the title and abstract of the studies. Two reviewers (Y.Y. Wu and P. Zhou) independently assessed the study quality using a revised tool for the quality assessment of diagnostic accuracy studies (QUADAS‐2) (Whiting et al., 2011). We tailored the guidelines for scoring each item of the checklist to our review. Disagreements were resolved by consensus or arbitration by a third reviewer (Y.K. Zhao).

### 2.3 Data extraction

A total of 2 reviewers (Y.Y. Wu and M. Waresi) extracted data from the relevant studies based on the criteria. Following data is independently extracted: authors' name, publication time, study design, culprit artery located through CAG. Extracted index test included STD in I for RCA, STD in aVL for RCA, STD in aVR for LCX, STD in V1 for LCX, STE in III > II for RCA, STD aVL > I for RCA, STD V3/STE III ≤ 1.2 for RCA, STE in V5 or V6 for LCX, sum (STD V1 + V2 + V3)/sum (STE II + III + aVF) ≤ 1 for RCA, STE III > II & STD aVL > I for RCA, JIM score >1.5 for LCX (Jim et al., 2012), Fiol algorithm for LCX (Fiol et al., 2004) and Tierala algorithm for LCX (Tierala et al., 2009). Other diagnostic methods were not extracted due to the small number of studies.

### 2.4 Data analysis

Pooled Sensitive, Specificity, positive likelihood ratio (PLR), negative likelihood ratio (NLR), and diagnostic odds ratio (DOR) were calculated for each index test. The pooled DOR is a single indicator of test performance that pooled measure of the performance of a diagnostic test. We used the bivariate model for analysis and pooling of the diagnostic performance measures. Forest plots with 95% confidence interval (CI) were calculated and depicted. We developed a bivariate summary receiver operating curve (sROC) and calculated the area under the curve (AUC) to examine the diagnostic accuracy of the diagnostic method containing five or more studies. Meta‐regression and publication bias were estimated in diagnostic test with 10 or more studies.

Statistical heterogeneity was assessed by applying the *Q* (significant if *p* < .1) and *I* ^2^ (significant if *I* ^2^ > 50%) statistic. *p* < .05 was considered to be statistically significant. Summary estimates of sensitivity and specificity and their 95% CI and forest plot, sROC curves were performed with R 4.1.2 (Shim et al., 2019). Publication bias, heterogeneity assessment, and meta‐regression analysis were performed with the STATA 17.0 software.

## 3 RESULTS

### 3.1 Study characteristics

**FIGURE 1:**
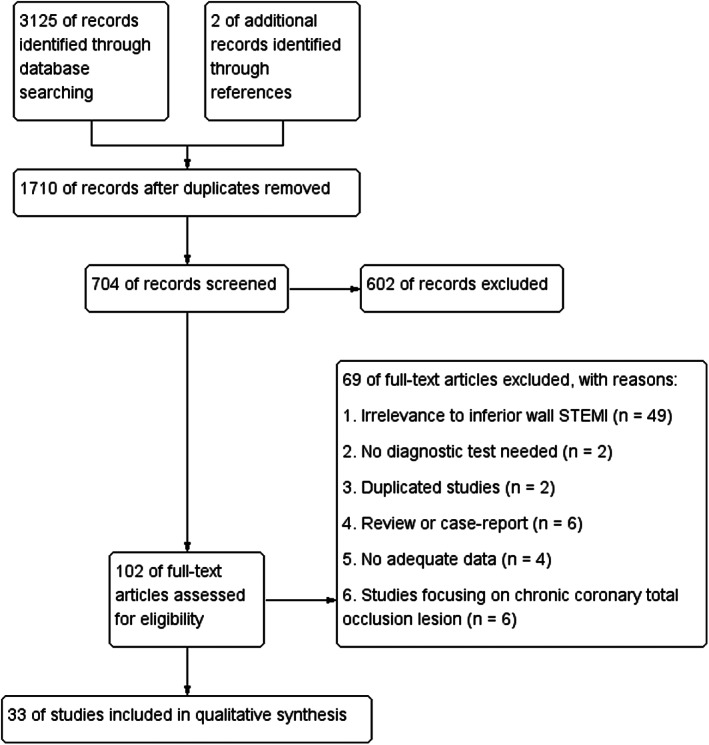
Flow diagram of search and study selection process for this review. STEMI, ST‐segment elevation myocardial infarction

**FIGURE 2:**
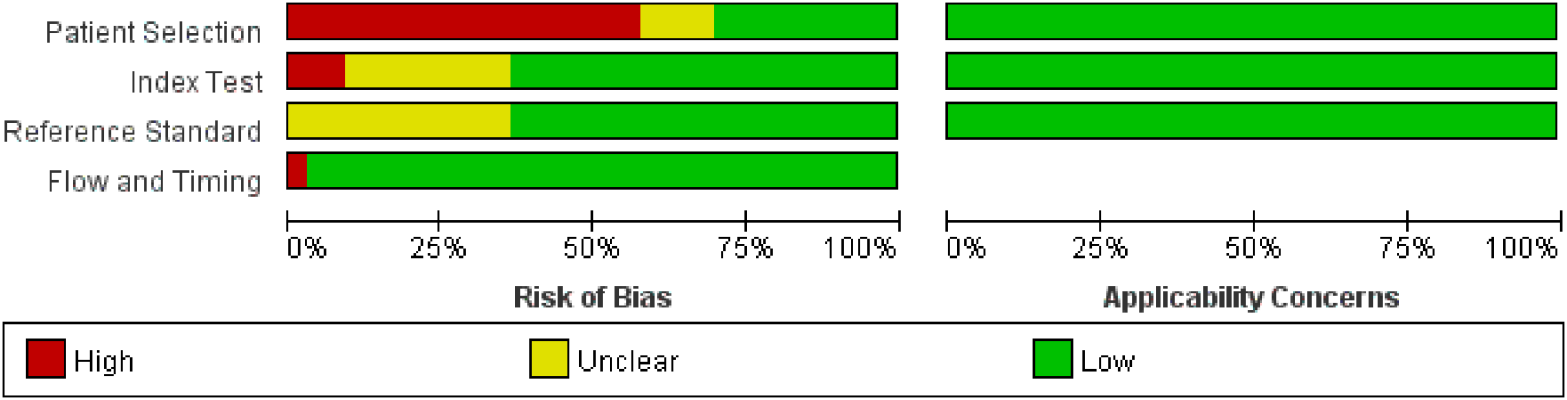
Risk of bias applicability concerns summary of each domain

Totally, 3127 records were retrieved after primarily searching literatures, while after screening the titles and abstracts, 602 records were excluded. A total of 102 potentially relevant full‐text articles were reviewed, and 33 studies with 4414 participants were included in the final analysis (Figure 1). Risk of bias was summarized in Figure 2. In patient selection domain, 19 out of 33 studies had a high bias risk, mainly because of a case–control design in 18 studies. In index test domain, 12 studies did not interpret the index test without blinding to the reference standard or did not mention that. In reference standard domain, 12 studies had unknown risk for not elucidating unawareness of the results of index test before interpreting the reference standard. In the flow and timing domain, one study had high risk for excluding patients from the analysis. All studies were assigned low concern regarding applicability. Study characteristics are listed in Table 1.

**TABLE 1:** The characteristics of enrolled studies

Author	Year	n	RCA	LCX	Lesions in non‐culprit arteries	ST measured from J point	Study design	Diagnostic methods and algorithms
Assali et al. (1999)	1999	83	66	17	−	80 ms	Retrospective	STE in V5 or V6 ≥ 1 mm
Gaballa et al. (2019)	2019	100	50	50	−	60 ms	Prospective	STD in aVR ≥1 mm, STE in III > II, STD in I
Mohanty & Saran (2016)	2016	100	74	26	−	80 ms	Prospective	STE in III ≥ II, STD in V3/STE in III ≤1.2, STD in aVL > I, sum of STD in V1‐3 < sum of STE in inferior leads
Chia et al. (2000)	2000	92	72	20	−	80 ms	Unknown	STE in III > II, STD in I
Ruiz‐Mateos et al. (2020)	2020	252	206	46	−	80 ms	Retrospective	STE in III > II
Bairey et al. (1987)	1987	41	29	12	−	80 ms	Retrospective	STE in V5 or V6
Chiang et al. (2006)	2006	40	32	8	−	80 ms	Retrospective	STD in I ≥ 1 mm, STD in aVL ≥1 mm, STE in III ≥ II, STD in aVL > I, STE in III ≥ II & STD in aVL ≥ I
Wong & Freedman (1996)	1996	95	77	18	−	60 ms	Retrospective	STE in V5 or V6, STD in V1 > 1 mm
Hasdai et al. (1995)	1995	62	46	16	−	60 ms	Retrospective	STD in I ≥ 1 mm, STD in aVL ≥1 mm
Bayram & Atalay (2004)	2004	73	53	20	−	80 ms	Retrospective	STE in III > II
Kabakci et al. (2001)	2001	149	123	26	−	60 ms	Unknown	STE in III > II, STD in aVL > I
Herz et al. (1997)	1997	83	66	17	−	80 ms	Retrospective	STE in V5 or V6 ≥ 1 mm, STD in I ≥ 1 mm, STD in aVL ≥1 mm, STE in III > II, STD in aVL > I, STE in III > II & STD in aVL > I
Tierala et al. (2009)	2009	98	84	14	+	60 ms	Prospective	Tierala algorithm, Fiol algorithm
Pourafkari et al. (2016)	2016	150	117	33	+	0 ms	Prospective	STD in aVR
Vales et al. (2011)	2011	106	83	23	+	60 ms	Retrospective	STD in aVR, Tierala algorithm, Fiol algorithm
Almansori et al. (2010)	2010	687	539	148	−	0 ms	Retrospective	STE in III > II, STD in V3/STE in III ≤1.2, STD in aVL > I, sum of STD in V1‐3 ≦ sum of STE in inferior leads, STD in I
Fiol et al. (2004)	2004	63	50	13	−	60 ms	Retrospective	STE in III > II, STD in I, sum of STD in V1‐3 ≤ sum of STE in inferior leads
Jim et al. (2012)	2012	78	64	14	−	80 ms	Prospective	STE in III > II, STD in V3/STE in III ≤1.2
Kontos et al. (1997)	1997	72	59	13	−	80 ms	Unknown	STD in I, STD in aVL, STD in V1, STE in V5 or V6
Kosuge et al. (1998)	1998	152	133	19	+	0 ms	Prospective	STD in V3/STE in III ≤1.2
Vives‐Borras et al. (2019)	2019	230	119	111	+	80 ms	Retrospective	STE in III > II, STD in V3/STE in III ≤1.2, STD in aVL > I, sum of STD in V1‐3 ≤ sum of STE in inferior leads, STD in I, STD in aVL ≥1 mm, STD in aVR, STE in V5 or V6, STD in V1, Tierala algorithm, Fiol algorithm
Taglieri et al. (2014)	2014	365	270	95	+	0 ms	Retrospective	STE in III > II, STD in I, sum of STD in V1‐3 ≤ sum of STE in inferior leads, Tierala algorithm, Fiol algorithm
Zimetbaum et al. (1998)	1998	69	52	17	−	80 ms	Prospective	STE in III > II
Li et al. (2011)	2011	75	59	16	−	80 ms	Retrospective	STE in III > II, STD in aVL > I, STD in I, STD in aVL, STD in aVR ≥1 mm, STD in V3/STE in III < 1.2, STE in V5 or V6
Li et al. (2017)	2017	240	177	63	−	80 ms	Retrospective	STE in III ≥ II, STD in aVL ≥ I, STD in I
Nair & Glancy (2002)	2002	30	25	5	−	60 ms	Prospective	STE in III > II, STD in aVR ≥1 mm
Baptista et al. (2004)	2004	53	38	15	−	60 ms	Retrospective	STE in III > II, STD in aVR, STD in V3/STE in III ≤1.2, STD in V1
Karbalaie et al. (2014)	2014	75	56	19	+	80 ms	Retrospective	STD in I, sum of STD in V1‐3 ≤ sum of STE in inferior leads, Fiol algorithm
Sun et al. (2007)	2007	90	70	20	−	60 ms	Prospective	STE in III > II, STD in aVL > I, STD in aVR ≥1 mm
Wong et al. (2004)	2004	177	150	27	+	80 ms	Unknown	STE in III > II, STD in I, STD in aVR
Huang et al. (2016)	2016	194	166	28	+	0 ms	Retrospective	STE in III > II, STD in aVL > I, STD in V3/STE in III ≤1.2, sum of STD in V1‐3 ≤ sum of STE in inferior leads, STD in I, STD in aVL, STD in aVR ≥1 mm, STE in V5 or V6 ≥ 1 mm, STD in V1
Kanei et al. (2010)	2010	105	86	19	+	60 ms	Prospective	STE in III > II, STD in I, STD in aVL, STD in aVR ≥1 mm, STD in V3/STE in III < 1.2
Zhong‐qun et al. (2009)	2009	135	117	18	+	0 ms	Prospective	STE in III > II, STD in aVL > I, STD in aVR ≥1 mm, STD in V3/STE in III ≤1.2

Abbreviations: STD, ST‐segment depression; STE, ST‐segment elevation.

### 3.2 Diagnostic methods with single lead

**FIGURE 3:**
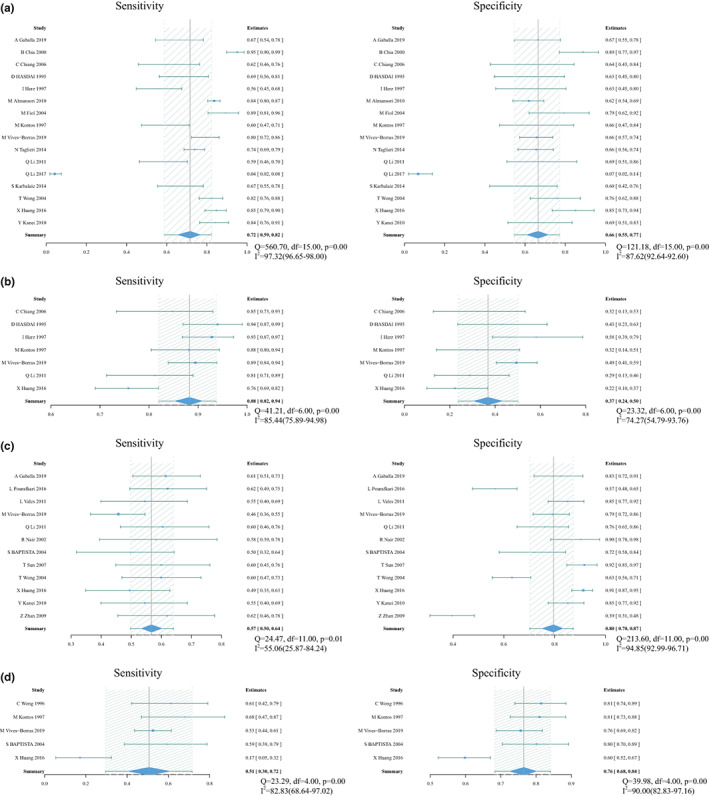
Forest plot of sensitivity and specificity and heterogeneity assessment for diagnostic methods using single lead. (a) Pooled sensitivity and specificity for ST‐segment depression (STD) in I for RCA. (b) Pooled sensitivity and specificity for STD in aVL for right coronary artery (RCA). (c) Pooled sensitivity and specificity for STD in aVR for left circumflex artery (LCX). (d) Pooled sensitivity and specificity for STD in V1 for LCX;Q and *I* ^2^ statistics for included studies suggested a high level of statistical heterogeneity. Solid squares = point estimate of each study (area indicates relative contribution of the study to meta‐analysis); horizontal lines = 95% confifidence interval (CI).

**FIGURE 4:**
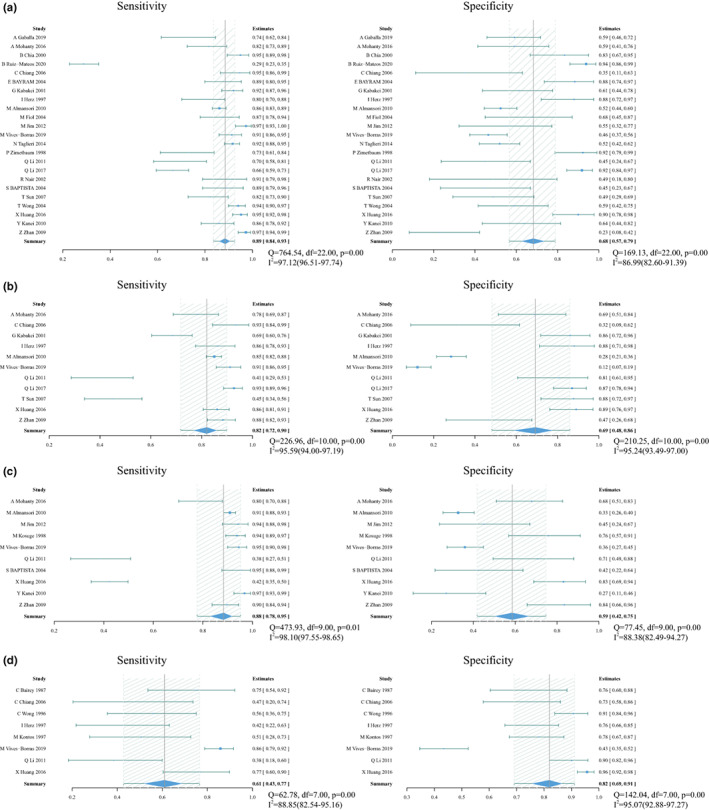
Summary receiver operating curve (sROC) of diagnostic methods. (a) SROC of diagnostic methods for RCA. (b) SROC of diagnostic methods for LCX. STD, ST‐segment depression; STE, ST‐segment elevation.

There were four index tests using single lead to predict culprit vessel, including STD in I for RCA (16 studies with 2660 patients), STD in aVL for RCA (7 studies with 756 patients), STD in aVR for LCX (12 studies with 1444 patients) and STD in V1 for LCX (5 studies with 638 patients). Forest plot of the study estimates of sensitivity and specificity for each test is shown in Figure 3, with sROC in Figure 4.

#### 3.2.1 STD in I

**FIGURE 5:**
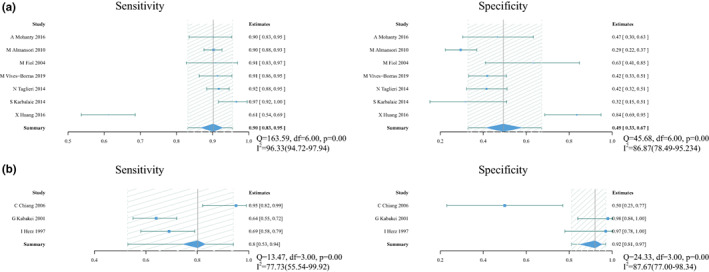
Funnel plots along with Egger tests for publication bias. (a) Funnel plots of ST‐segment depression (STD) in I. (b) Funnel plots of STD in aVR. (c) Funnel plots of ST‐segment elevation (STE) in III > II. (d) Funnel plots of STD in aVL > I. (e) Funnel plots of STD in V3/STE in III ≤1.2. No significant publication bias existed in the analysis.

Pooled sensitivity and specificity were 0.72 (0.57–0.84) and 0.66 (0.52–0.78), respectively. Pooled PLR and NLR were 2.1 (1.2–3.7) and 0.42 (0.22–0.81), respectively. DOR was 5 (2–17) and AUC was 0.74 (0.70–0.78). For the heterogeneity, covariates were added to our bivariate model to explore the source of heterogeneity (Table 2). However, these added covariates did not change the heterogeneity among these studies. No significant publication bias existed in the data (*p* .61, Figure 5).

**TABLE 2:** *p* value of meta‐regression

	STD in I		STD in aVR		STE in III > II		STD in aVL > I		STD in V3/STE in III ≤1.2	
	Sen	Spec	Sen	Spec	Sen	Spec	Sen	Spec	Sen	Spec
Study design	0.69	0.99	0.23	0.31	0.11	0.16	0.17	0.79	0.26	0.71
Cut‐off point	0.46	0.96	0.65	0.89	0.30	0.85	0.15	0.08	0.32	0.45
Multivessel disease	0.68	1.00	0.12	0.02*	0.56	0.26	0.77	0.26	0.69	0.50
ST measured from J point	0.09	0.34	0.35	0.17	0.01*	0.37	0.97	0.65	0.41	0.68

Abbreviations: Sen, sensitivity; Spec, specificity; STD, ST‐segment depression; STE, ST‐segment elevation.

* *p* < .05.

#### 3.2.2 STD in aVL

Pooled sensitivity and specificity were 0.88 (0.80–0.93) and 0.37 (0.24–0.54), respectively. Pooled PLR and NLR were 1.4 (1.1–1.9) and 0.32 (0.15–0.71), respectively. DOR was 4 (2–13) and AUC was 0.75 (0.71–0.79).

#### 3.2.3 STD in aVR

Pooled sensitivity and specificity were 0.57 (0.50–0.64) and 0.80 (0.70–0.87), respectively. Pooled PLR and NLR were 2.8 (1.8–4.4) and 0.54 (0.44–0.66), respectively. DOR was 5 (3–10) and AUC was 0.67 (0.63–0.71). Meta‐regression showed that multivessel disease contributed to the heterogeneity of specificity (Table 2). No significant publication bias existed in the data (*p* .18, Figure 5).

#### 3.2.4 STD in V1

Pooled sensitivity and specificity were 0.51 (0.30–0.72) and 0.76 (0.68–0.84), respectively. Pooled PLR and NLR were 2.2 (1.0–4.8) and 0.63 (0.35–1.13), respectively. DOR was 4 (1–14) and AUC was 0.75 (0.71–0.78).

### 3.3 Diagnostic methods with double leads

**FIGURE 6:**
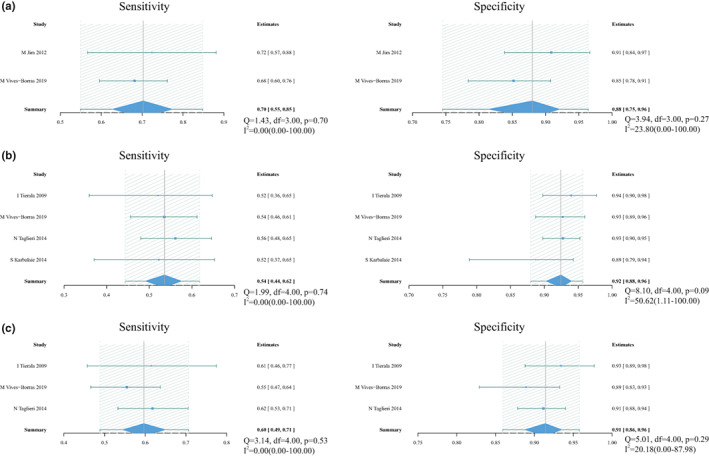
Forest plot of sensitivity and specificity and heterogeneity assessment for diagnostic methods using double leads. (a) Pooled sensitivity and specificity for ST‐segment elevation (STE) in III > II for right coronary artery (RCA). (b) Pooled sensitivity and specificity for ST‐segment depression (STD) in aVL > I for RCA. (c) STD in V3/STE in III ≤1.2 for RCA. (d) STE in V5 or V6 for left circumflex artery; *Q* and *I* ^2^ statistics for included studies suggested a high level of statistical heterogeneity. Solid squares = point estimate of each study (area indicates relative contribution of the study to meta‐analysis); horizontal lines = 95% confifidence interval (CI).

There were four index tests using double leads to predict culprit vessel, including STE in III > II for RCA (23 studies with 2660 patients), STD in aVL > I for RCA (11 studies with 2023 patients), STD V3/STE III ≤1.2 for RCA (10 studies with 1809 patients) and STE in V5 or V6 for LCX (eight studies with 830 patients). Forest plot is shown in Figure 6, with sROC in Figure 4.

#### 3.3.1 STE in III > II

Pooled sensitivity and specificity were 0.89 (0.84–0.93) and 0.68 (0.57–0.79), respectively. Pooled PLR and NLR were 2.8 (1.9–4.2) and 0.16 (0.11–0.25), respectively. DOR was 17 (9–32) and AUC was 0.88 (0.85–0.91). Meta‐regression showed that the position of ST‐segment measured from J point contributed heterogeneity to sensitivity (Table 2). No significant publication bias existed in the data (*p* .2, Figure 5).

#### 3.3.2 STD in aVL > I

Pooled sensitivity and specificity were 0.82 (0.72–0.90) and 0.69 (0.48–0.86), respectively. Pooled PLR and NLR were 2.8 (1.4–5.5) and 0.25 (0.15–0.41), respectively. DOR was 11 (4–29) and AUC was 0.85 (0.81–0.88). Added covariates did not change the heterogeneity in meta‐regression. No significant publication bias existed in the data (*p* .21, Figure 5).

#### 3.3.3 STD V3/STE III ≤1.2

Pooled sensitivity and specificity were 0.88 (0.78–0.95) and 0.59 (0.42–0.75), respectively. Pooled PLR and NLR were 2.2 (1.5–3.2) and 0.18 (0.09–0.38), respectively. DOR was 12 (5–27) and AUC was 0.82 (0.78–0.85). Added covariates did not change the heterogeneity in meta‐regression. No significant publication bias existed in the data (*p* .18, Figure 5).

#### 3.3.4 STE in V5 or V6

Pooled sensitivity and specificity were 0.61 (0.43–0.77) and 0.82 (0.69–0.91), respectively. Pooled PLR and NLR were 3.5 (1.8–6.8) and 0.47 (0.30–0.74), respectively. DOR was 8 (3–20) and AUC was 0.79 (0.75–0.82).

### 3.4 Diagnostic methods with multilead

**FIGURE 7:**
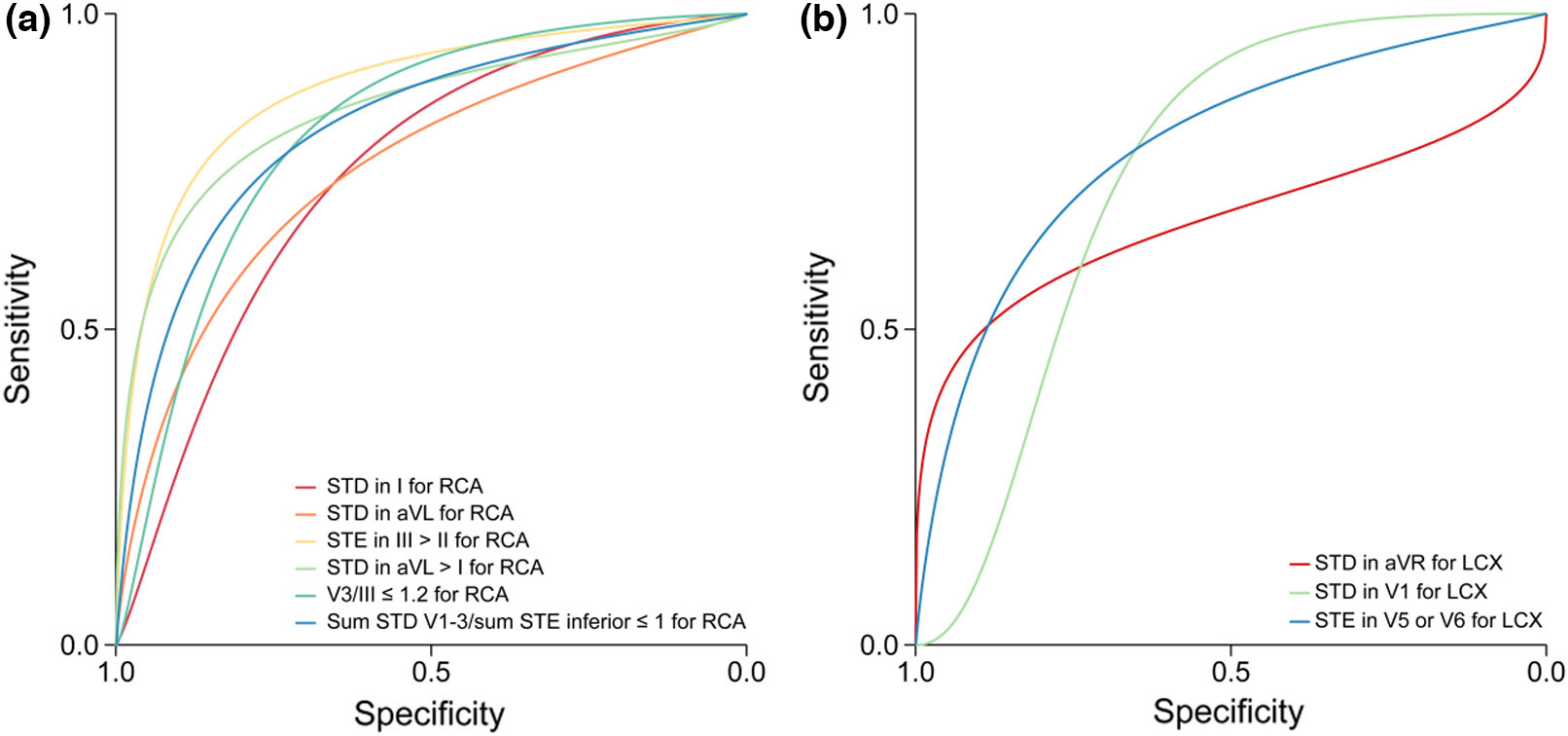
Forest plot of sensitivity and specificity and heterogeneity assessment for diagnostic methods using multilead. (a) Pooled sensitivity and specificity for Sum (ST‐segment depression V1 + V2 + V3)/sum (ST‐segment elevation II + III + aVF) ≤ 1 for right coronary artery (RCA). (b) Pooled sensitivity and specificity for ST‐segment elevation in III > II & ST‐segment depression in aVL > I for RCA; *Q* and *I* ^2^ statistics for included studies suggested a high level of statistical heterogeneity. Solid squares = point estimate of each study (area indicates relative contribution of the study to meta‐analysis); horizontal lines = 95% confifidence interval (CI).

There were two index tests using multilead to predict culprit vessel, including sum (STD V1 + V2 + V3)/sum (STE II + III + aVF) ≤ 1 for RCA (7 studies with 1714 patients) and STE in III > II & STD in aVL > I for RCA (three studies with 315 patients). Forest plot is shown in Figure 7, with sROC in Figure 4.

#### 3.4.1 Sum (STD V1 + V2 + V3)/sum (STE II + III + aVF) ≤ 1

Pooled sensitivity and specificity were 0.90 (0.83–0.95) and 0.49 (0.33–0.67), respectively. Pooled PLR and NLR were 1.8 (1.3–2.4) and 0.20 (0.13–0.33), respectively. DOR was 9 (5–16) and AUC was 0.82 (0.79–0.85).

#### 3.4.2 STE in III > II & STD in aVL > I

Pooled sensitivity and specificity were 0.80 (0.53–0.94) and 0.92 (0.81–0.97), respectively.

### 3.5 Diagnostic algorithms

**FIGURE 8:**
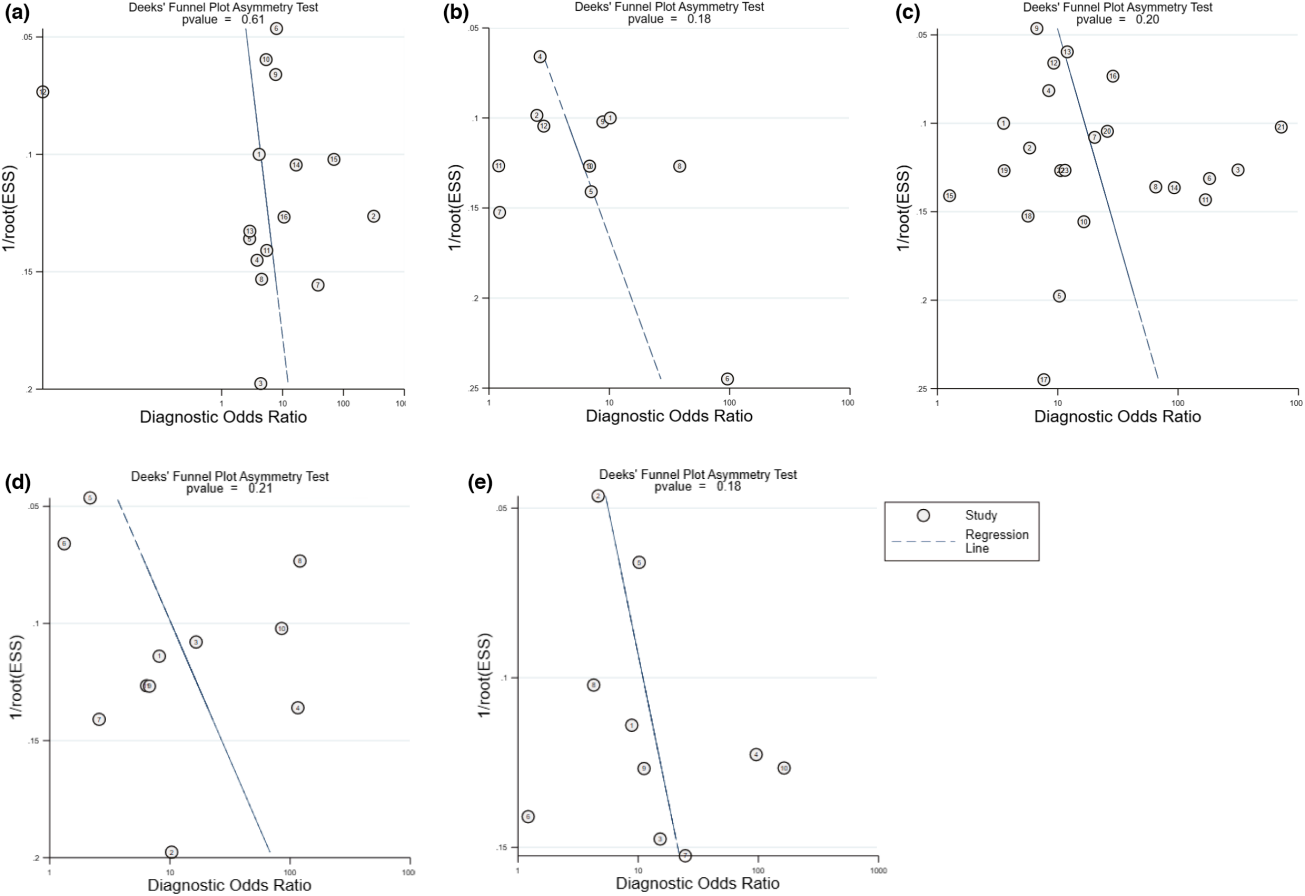
Forest plot of sensitivity and specificity and heterogeneity assessment for diagnostic algorithms. (a) Pooled sensitivity and specificity for Jim score >1.5 for left circumflex artery (LCX). (b) Pooled sensitivity and specificity for Fiol's algorithm for LCX. (c) Pooled sensitivity and specificity for Tierala's algorithm for LCX; *Q* and *I* ^2^ statistics for included studies suggested a low level of statistical heterogeneity. Solid squares = point estimate of each study (area indicates relative contribution of the study to meta‐analysis); horizontal lines = 95% confifidence interval (CI).

There were three algorithms to predict culprit vessel, including Jim score >1.5 for LCX (2 studies with 308 patients), Fiol's algorithm for LCX (4 studies with 768 patients) and Tierala's algorithm for LCX (3 studies with 693 patients). Forest plot is shown in Figure 8.

Jim score: Pooled sensitivity and specificity were 0.70 (0.55–0.85) and 0.88 (0.75–0.96), respectively.

Fiol's algorithm: Pooled sensitivity and specificity were 0.54 (0.44–0.62) and 0.92 (0.88–0.96), respectively.

Tierala's algorithm: Pooled sensitivity and specificity were 0.60 (0.49–0.71) and 0.91 (0.86–0.96), respectively.

## 4 DISCUSSION

After three decades of study, there were some achievements in identifying culprit artery using ECG in inferior wall STEMI. However, these diagnostic methods were not widely used. There is still a lack of comparation on diagnostic properties among these methods. This study indicated that several methods could help identify the culprit artery.

On the basis of DOR and AUC, methods using double leads have better properties than single lead (Søreide et al., 2011). Among these methods, STE in III > II has the highest AUC and was the most widely used method, which can be the cornerstone of diagnosis. Although STD in V3/STE in III ≤ 1.2 was a satisfying method considering its parameters similar to STD in aVL > I, a higher convenience of the latter one made it a more ideal method. We presumed that STE in III > II together with STD in aVL > I might be the most valuable method. Unfortunately, it was only involved in three studies, in which high sensitivity and specificity were exhibited.

A surprising finding was that previous diagnostic algorithms were not superior to these simple methods. In the original studies, these algorithms all exhibited extremely high sensitivity and specificity, even nearly 100% (Fiol et al., 2004; Tierala et al., 2009). However, this property was not proved in population derived from other studies. It was partly because the case–control design of these original studies caused higher risk of bias. These algorithms derived from specific populations had lower external validity (Song & Chung, 2010). In addition, the algorithms involved three or four steps with at least five leads. The complicacy determined their unpopularity, especially in emergency condition, in which the shorter door‐to‐balloon time was the better (Namdar et al., 2021).

We observed that heterogeneity existed in most diagnostic methods. We considered four possible reasons, including study design, cut‐off point, the distance from J point to measured ST‐segment, multivessel disease. For this study, study design did not change the heterogeneity. Probable reason was that ECG was an objective measure. Similarly, cut‐off point did not affect heterogeneity. A small number of sample size for different cut‐off points might account for this (Huang et al., 2016). Previous study indicated that different algorithms to detect the culprit artery in inferior STEMI patients can change significantly depending on the point where ST elevation or depression is measured (Ruiz‐Mateos et al., 2019). In this review, this distance did not affect heterogeneity as well. The simple methods might have less observational bias. Multivessel disease may affect ECG findings and even interfere the identification of culprit artery (Yildirimturk et al., 2020). Meta‐regression proved its effect.

Some other methods which were not included in this study might also help identify the culprit artery. T‐wave inversion in lead I might be a sign of RCA related infarction (Wong et al., 2004). S/R‐wave ratio < 1/3 with ST depression <1 mm indicated LCX‐related infarction (Assali et al., 1999). STD in I = 1/2 aVL for RCA and STE in II = III = aVF for LCX was regarded as another algorithm (Huang et al., 2016). However, few studies investigated the effectiveness of these methods.

Ravi et al. (2011) developed two ECG algorithms trying to identify proximal RCA, proximal LCX, or distal RCA or LCX in inferior wall STEMI. In these algorithms, STE in II > III and no STD in aVL represents proximal LCX occlusion, while STE in III > II and no STD in V1 represents proximal RCA occlusion. Differences in the magnitude of STD or T‐wave direction are considered to indicate proximal LCX occlusion. However, positive and negative predictive values of these algorithms were low. These ECG algorithms cannot reliably identify the culprit artery in inferior wall STEMI.

This meta‐analysis showed the diagnostic value of ECG in identifying culprit artery in inferior wall STEMI. Moreover, ECG is also valuable in identifying the infarct‐related artery in patients with inferior wall STEMI with critical lesions in both the RCA and the LCX (Yildirimturk et al., 2020). However, most diagnostic methods lose their strength in this condition.

This study had some limitations. Firstly, although there were over 30 studies included, the sample size was relatively small. The diagnostic properties of STE in III > II & STD in aVL > I and algorithms needed verifying. With more research, a standard diagnostic method might be developed. Secondly, coronary artery anomalies are common in clinical practice (Tomanek & Angelini, 2019). Most diagnostic methods are accurate only when RCA is the dominant artery, though right dominance is more common (Taglieri et al., 2014). Only through angiograph doctors can recognize the anatomy and localize the precise lesion.

## 5 CONCLUSION

Several diagnostic methods using 12‐lead ECG can help localize the culprit artery in inferior wall STEMI before primary PCI allowing immediate decisions about therapy. Our meta‐analysis indicated that diagnostic methods using double leads had better properties. STE in III > II together with STD in aVL > I may be the most ideal method, for its accuracy and convenience.

## AUTHOR CONTRIBUTIONS

J. Li, B. Jin and X.P. Luo designed the study; Y.J. Yu, Y.K. Zhao and M. Wang searched the studies; Y.Y Wu, M. Waresi and H.Y. Li screened articles, extracted data, assessed the risk of bias and analyzed data; P. Zhou wrote the manuscript.

## FUNDING INFORMATION

This study received no specific grant from any funding agency in the public, commercial, or not‐for‐profit sectors.

## CONFLICT OF INTEREST

The authors declare no conflicts of interest.

## 6 ETHICAL APPROVAL

This meta‐analysis was approved by the Ethics and Research Committees of Huashan Hospital, Fudan University.

## Data Availability

Data sharing not applicable to this article as no datasets were generated or analysed during the current study.
